# *in vivo* analysis of active versus self-tapping implants with SLA surface treatment

**DOI:** 10.6026/973206300213152

**Published:** 2025-09-30

**Authors:** Bandaru Archana, Vidhyadhar D., Sravanthi E., Guru Charan Karthik K.V., Prathyusha Pulleti, Kiran Kunwar Rathore

**Affiliations:** 1 Department of Prosthodontics, CKS Teja Institute of Dental Sciences and Research, Tirupathi andhar Pradesh, India; 2 Department of Prosthodontics and crown & bridge, Private practitioner ,Hyderabad, Telangana, India; 3 Department of Prosthodontics, Army College of Dental Sciences, Secunderabad,Telangana, India; 4 Department of Prosthodontics and Crown & Bridge, Army College of Dental Sciences, Secunderabad, Telangana, India; 5 Department of Dental Surgery, MPS Health Informatics, University of Maryland, Baltimore County United States of America; 6 Department of Prosthodontics, Army College of Dental sciences, Secunderabad, Telangana, India

**Keywords:** Dental implants, SLA surface, active-threaded, self-tapping, osseointegration, early loading, crestal bone loss, peri-implant evaluation

## Abstract

Surface modifications and implant thread designs play a crucial role in achieving primary stability and osseointegration. Therefore, it is of interest to compare the short-term clinical and radiographic outcomes of active versus self-tapping titanium implants with sandblasted, large-grit, acid-etched (SLA) surfaces in 20 systemically healthy patients requiring posterior implant-supported prostheses. Clinical and radiographic parameters were assessed at 4, 7, 12 and 26 weeks and statistical analysis showed significant intra-group improvements in probing depth, attachment levels, mucosal margin stability and bone gain. However, intergroup comparisons at all-time points revealed no statistically significant differences, indicating comparable performance between the two implant designs. Overall, both active and self-threaded SLA implants demonstrated favorable short-term clinical outcomes, supporting their effectiveness in promoting early osseointegration and potential use in early loading protocols.

## Background:

Modern dentistry strives to restore contour, function, esthetics, speech and overall oral health. Among the available treatment modalities, implant dentistry plays a pivotal role in achieving these goals, particularly in patients with partial or complete edentulism. Technological advancements in implant materials, diagnostics, surgical techniques and surface modifications have enhanced treatment outcomes, even in complex clinical scenarios [1]. Initially, implant therapy success was measured primarily by survival rates. However, contemporary success criteria emphasize peri-implant tissue health, prosthesis stability, patient satisfaction and minimal bone loss [2]. A critical determinant of long-term success is osseointegration, which can be improved by modifying implant surface topography rather than altering base material composition [3]. Surface treatments, especially sand-blasted, large-grit, acid-etched (SLA) techniques, significantly enhance implant roughness, promoting better cell adhesion, proliferation and bone integration [4]. Implants with modified SLA surfaces have demonstrated faster healing, reducing osseointegration periods from 3-6 months to 6-8 weeks [5]. In parallel, immediate or early loading protocols have gained popularity, reducing patient discomfort associated with prolonged healing and use of removable prostheses [6]. Additionally, thread design plays a crucial role in improving primary stability, optimizing load distribution and supporting long-term success [7]. These innovations necessitate further evaluation of implant systems with different thread geometries and surface treatments to optimize early loading protocols. Therefore, it is of interest to report the short-term clinical and radiographic performance of titanium screw-type implants with chemically modified SLA surfaces following a three-week healing period.

## Materials and Methods:

This prospective *in vivo* study was conducted in the Department of Prosthodontics, CKS Theja Dental College and Research Institute, Tirupati, following ethical approval from the Institutional Review Board. The minimum required sample size was estimated based on previous studies evaluating implant stability and crestal bone changes in SLA-surfaced implants. Assuming a clinically significant difference of 1.0 mm in bone level between groups, with a standard deviation of 0.8 mm, a power of 80% and a significance level of 0.05, a sample size of 8 subjects per group was calculated. To account for potential dropouts and improve statistical robustness, the sample size was rounded to 10 implants per group, totaling 20 implants. Thus, 20 patients were enrolled, equally distributed between the active and self-threaded implant groups. A total of 20 medically fit patients, aged between 20 and 50 years, requiring implant-supported prostheses in posterior edentulous sites were enrolled after obtaining written informed consent. The participants were randomly divided into two groups. Group A received Active Threaded implants with a Sandblasted, Large grit and Acid-Etched (SLA) surface (HI-TEC® Implants, LifeCare Devices Pvt. Ltd., Israel) and Group B received Self-Threaded SLA implants of the same brand. Each group was further subdivided based on the loading protocol-occlusal or non-occlusal-based on individual case criteria and primary stability achieved. Preoperative assessments included detailed case histories, hematological evaluations, orthopantomograms (OPG) and diagnostic casts. Custom-made radiographic stents embedded with radiopaque markers were fabricated using clear acrylic resin (DPI^TM^, Mumbai, India) to ensure standardized radiographic positioning during follow-up evaluations. All implant surgeries were performed under strict aseptic conditions. After administration of 2% lignocaine with 1:80, 000 adrenalines, a mid-crestal incision with releasing incisions was made, followed by elevation of a full-thickness mucoperiosteal flap.

Osteotomy site preparation was done as per the manufacturer's surgical protocol using graduated drills under copious sterile saline irrigation. Implants were placed 1 mm subcrestally. The insertion torque was measured to ensure primary stability and healing abutments were immediately placed. Postoperative care included systemic antibiotics (amoxicillin 500 mg TID for 5 days), analgesics (ibuprofen 400 mg BID for 3 days) and 0.12% chlorhexidine mouth rinse for 2 weeks. Sutures were removed after 7-10 days. In the early loading groups, provisional acrylic crowns were placed without occlusal contact at 2 weeks. In delayed loading cases, definitive prosthetic loading was done after 3 months. Final impressions were made using addition silicone (Aquasil Soft Putty, Dentsply Sirona), poured with Type IV dental stone (Kalrock, Kalabhai, India). Custom abutments and porcelain-fused-to-metal (PFM) crowns were fabricated and cemented using glass ionomer cement. Follow-up evaluations were carried out at baseline, 4, 7, 12 and 26 weeks. Clinical indices assessed included the Modified Plaque Index (Mombelli *et al.* 1987), Sulcus Bleeding Index (Löe & Silness, 1963), Probing Pocket Depth and Clinical Attachment Level. Standardized intraoral periapical radiographs were captured using the long-cone paralleling technique. Digital images were analyzed using Adobe Photoshop CS6 to measure the distance between the implant shoulder and the first bone-to-implant contact (DIB) [2, 5]. All radiographs were calibrated using a reference implant length for standardization. Measurements were made by a single experienced examiner to reduce observer bias. Data were analyzed using SPSS Version 23.0 (IBM Corp., Armonk, NY, USA). Descriptive statistics were used to summarize the data. Wilcoxon Signed-Rank Test assessed intra-group differences, while the Mann-Whitney U Test compared inter-group variations. A p-value < 0.05 was considered statistically significant.

## Results:

Clinical parameters were assessed at 4th, 7th, 12th and 26th weeks to evaluate peri-implant soft and hard tissue changes over time. A statistically significant increase in probing depth was observed (p = 0.012), with mean values rising from 0.47± 0.43 mm at 4 weeks to 0.96 ± 0.48 mm at 26 weeks, indicating progressive soft tissue remodeling. Although DIM (Distance from Implant shoulder to Mucosal margin) showed a decreasing trend from -4.05 ± 0.78 mm to -2.08 ± 2.21 mm, the change was not statistically significant (p = 0.074), suggesting minimal variation in mucosal margin position. Attachment level demonstrated a significant improvement (p = 0.005), shifting from -2.92 ± 2.36 mm at 4 weeks to -1.85 ± 1.10 mm at 26 weeks, reflecting favorable soft tissue response and healing. DIB (Distance from Implant shoulder to Bone crest) also showed a significant increase (p = 0.005), progressing from 0.58 ± 0.61 mm to 1.48 ± 0.55 mm over the study period, suggesting ongoing crestal bone remodeling around the implant site (Table 1 - see PDF). Table 2 (see PDF) shows the longitudinal evaluation of peri-implant parameters across four time points (4th, 7th, 12th and 26th weeks) revealed notable changes. Probing depth showed a statistically significant increase over time (p = 0.011), rising from 0.46 ± 0.56 mm at 4 weeks to 1.06 ± 0.79 mm at 26 weeks, indicating progressive soft tissue changes possibly related to initial tissue remodeling. DIM (Distance from the Implant shoulder to the Mucosal margin) demonstrated a significant reduction (p = 0.005), decreasing from -3.30 ± 1.24 mm to -1.99 ± 0.94 mm, reflecting coronal migration of the mucosal margin or soft tissue maturation. Attachment level showed fluctuations without statistical significance (p = 0.074), suggesting relative stability in connective tissue attachment over time. In contrast, DIB (Distance from Implant shoulder to Bone crest) increased significantly (p = 0.007), from 0.60 ± 0.57 mm to 2.03 ± 1.06 mm, indicating ongoing crestal bone remodeling or resorption around the implant. These findings highlight both soft and hard tissue dynamics during the early healing phase, with probing depth and crestal bone changes warranting careful long-term monitoring. The intragroup analysis of peri-implant parameters at various follow-up intervals (4th, 7th, 12th and 26th weeks) demonstrated no statistically significant differences over time. Changes in probing depth across the time points were minimal, with mean differences ranging from -0.09 to 0.09 mm and all p-values >0.7, indicating no significant alteration in soft tissue probing measurements. Similarly, DIM (distance from implant shoulder to mucosal margin) showed non-significant fluctuations, including a slight increase at the 26th week (mean difference = 0.96 mm; p = 0.362). Attachment level also remained relatively stable, with changes ranging from -0.72 to 0.37 mm and all comparisons being statistically non-significant (p > 0.4). DIB (distance from implant shoulder to bone crest) showed a trend towards increased values by the 26th week (mean difference = -0.55 mm), possibly indicating crestal bone remodeling; however, this change was not statistically significant (p = 0.100). Overall, the data suggest that while there were minor variations in peri-implant soft and hard tissue measurements over time, none reached statistical significance, pointing to a period of relative clinical stability during the follow-up phase (Table 3 - see PDF).

## Discussion:

Tooth loss, often resulting from caries, trauma, or periodontal disease, significantly impairs oral function. Traditional options like removable dentures lack stability, while fixed bridges compromise adjacent healthy teeth. Dental implants offer a fixed, self-supporting solution with high success rates (~97%), preserving adjacent structures [8]. Crestal bone plays a pivotal role in implant longevity. Early implant failure often begins at the crestal region due to bacterial colonization and peri-implantitis, affecting both function and aesthetics [9]. Bone loss can lead to papillary collapse, reduced load-bearing capacity and compromised gingival contours [10]. To mitigate crestal bone loss, innovations in implant design-such as sandblasting, acid-etching, thread modifications and scalloped platforms have been developed to enhance osseointegration and long-term success. Crestal bone loss is a recognized physiological response following second-stage implant surgery. Adell *et al.* reported an average of 1.2 mm bone loss around submerged two-piece implants in the first year [11], while Weber *et al.* found a 2.92 mm distance from the implant top to crestal bone, supporting the role of bone remodeling for biological width [12]. Similar findings were observed by Herman and Warren, who noted bone stabilization after an initial loss of 1.5-2 mm post-loading [13]. Titanium's high biocompatibility and corrosion resistance stem from its TiO_2_ layer. Grade 5 titanium alloy (Ti6Al4V), commonly used in implants, offers superior strength, fatigue resistance and lower elastic modulus compared to pure titanium, minimizing stress shielding [3]. Surface treatments-mechanical, chemical, or physical-enhance surface topography and energy, promoting osseointegration. Techniques such as SLA (sandblasting with large grit followed by acid etching) create micro- and macro-roughness to improve tissue integration [8].

The present study evaluated the early loading of SLA-modified titanium implants and demonstrated favorable short-term outcomes, with low risk of early failure and good cost-effectiveness. Present study shows significant intra-group improvements in probing depth, mucosal attachment and bone contact from 4 to 26 weeks in both active and self-threaded implant groups. However, no significant inter-group differences were observed, indicating both implant designs performed comparably. Implant stability initially relies on a press-fit mechanism (primary contact) with the surrounding bone, which transitions to secondary contact as healing progresses and woven bone is replaced by lamellar bone. Although the exact time at which an implant can reliably withstand functional torque forces (e.g., 35 Ncm) is still undetermined, the presence and quality of cortical and cancellous bone remain critical for long-term stability and resistance to micromovement [14]. Misch's bone classification (D1-D5) and Lekholm & Zarb's types (1-4) categorize bone quality based on cortical and trabecular composition, which significantly impacts implant stability and survival rates [15]. In this study, implants were placed in Type 1 to 3 bones, ensuring primary stability. Two-part implant systems, commonly used, minimize infection risk, while one-piece systems, though convenient, may lead to marginal bone loss due to bacterial infiltration [13]. Regardless of the system, maintaining crestal bone levels is key to implant success. Implant design-especially surface treatment and thread geometry-plays a crucial role in osseointegration. Sandblasted and acid-etched (SLA) surfaces enhance osteoblast attachment and allow for early loading within six weeks [15]. Thread depth significantly improves stability and stress distribution, particularly in softer bone, while pitch and face angle have less influence [3]. Together, these design factors optimize load transfer, initial fixation and long-term implant performance. Implant thread design significantly influences primary stability and long-term osseointegration. Key parameters such as thread pitch, depth and shape directly affect load distribution at the bone-implant interface. A smaller thread pitch increases the number of threads, enhancing surface area and load dissipation-particularly beneficial in cases with poor bone quality, short implants, or high occlusal forces, although it may complicate implant placement [16]. Deeper threads enhance fixation in trabecular bone by increasing bone contact, while their effect is less in dense cortical bone Misch. Thread shape also affects implant performance-square threads yield higher bone-implant contact (BIC) and reduce shear stress, whereas V-shaped and buttress designs may increase harmful shear forces [17]. Surface modifications, such as sandblasted, large-grit, acid-etched (SLA) or oxidized treatments, further improve early BIC and bone healing. Studies show oxidized implants demonstrate greater removal torque than SLA surfaces at 4 weeks, although differences equalize by 12 weeks [18].

These surface treatments enhance early osseointegration, increase bonding strength and support higher failure loads. Implant stability quotient (ISQ) values typically dip during initial bone remodeling but recover by 3 weeks and increase at 4 weeks, indicating progressive stabilization with SLA-treated surfaces [19]. Peri-implant probing is essential for diagnosing peri-implantitis and monitoring treatment outcomes, with increased probing depth and loss of clinical attachment considered key indicators of disease. Probing provides insights into the mucosal margin level, peri-implant pocket depth, tissue resistance and signs of inflammation such as bleeding or suppuration [20]. Unlike natural teeth, which have perpendicular connective tissue fibers inserting into cementum, implants lack this structural insertion, resulting in parallel fiber orientation and reduced resistance to probe penetration. This anatomical difference underscores the need for a standardized probing force-ideally around 0.25 N-for reliable measurements. Probing remains a simple, immediate and topographically informative diagnostic tool. Accurate assessment should consider attachment levels relative to fixed implant landmarks, as marginal recession may cause underestimation of bone loss if only pocket depth is evaluated. However, the present study's reliance on IOPA radiographs limited assessment to mesial and distal aspects and conventional imaging may lack sensitivity to early bone changes. Future studies should incorporate longer follow-up durations and advanced imaging like CBCT to evaluate all implants surfaces comprehensively. Clinically, surface-treated implants demonstrate superior osseointegration and regular, detailed evaluations are crucial for early detection of failure, allowing timely intervention and improved implant success.

## Conclusion:

Chemically modified roughened implant surfaces play a beneficial role in promoting early osseointegration is shown. These surface treatments appear to stimulate favorable biological responses at the implant-tissue interface, enhancing the initial stages of healing. Improved early integration may support the possibility of reducing the healing period before functional loading. Consequently, such advancements could lead to more predictable clinical outcomes and broaden the scope of implant placement protocols in contemporary dental practice.

## References

[1] Sartoretto SC (2023). J Funct Biomater..

[2] van Velzen FJ (2015). Clin Oral Implants Res..

[3] Ogle OE (2015). Dent Clin North Am..

[4] Kligman S (2021). J Clin Med..

[5] Bornstein MM (2009). Clin Implant Dent Relat Res..

[6] Gapski R (2003). Clin Oral Implants Res..

[7] Balshi TJ (2011). J Prosthodont..

[8] Duong HY (2022). Periodontol 2000..

[9] Lee D (2023). Clin Implant Dent Relat Res..

[10] Niznick G (1991). Compendium..

[11] Adell R (1981). Int J Oral Surg..

[12] Weber HP, Buser D (1996). Clin Oral Implants Res..

[13] Hermann JS (2000). J Periodontol..

[14] Cochran DL (2002). Clin Oral Implants Res..

[15] Palomino-Zorrilla JJ (2024). J Int Soc Prev Community Dent..

[16] Huang YC (2023). J Dent Sci..

[17] Menini M (2020). Biomed Res Int..

[18] Kim YH (2003). Int J Oral Maxillofac Implants..

[19] Kim JM (2009). J Adv Prosthodont..

[20] Narula S (2012). J Adv Oral Res..

